# Toxin-Induced Experimental Models of Learning and Memory Impairment

**DOI:** 10.3390/ijms17091447

**Published:** 2016-09-01

**Authors:** Sandeep Vasant More, Hemant Kumar, Duk-Yeon Cho, Yo-Sep Yun, Dong-Kug Choi

**Affiliations:** Department of Biotechnology, College of Biomedical and Health Science, Konkuk University, Chungju 27478, Korea; sandeepbcp@gmail.com (S.V.M.); hemantbhave@gmail.com (H.K.); ejrdus1026@naver.com (D.-Y.C.); aaa003132@naver.com (Y.-S.Y.)

**Keywords:** animal model, cognition, dementia, learning, memory, toxin

## Abstract

Animal models for learning and memory have significantly contributed to novel strategies for drug development and hence are an imperative part in the assessment of therapeutics. Learning and memory involve different stages including acquisition, consolidation, and retrieval and each stage can be characterized using specific toxin. Recent studies have postulated the molecular basis of these processes and have also demonstrated many signaling molecules that are involved in several stages of memory. Most insights into learning and memory impairment and to develop a novel compound stems from the investigations performed in experimental models, especially those produced by neurotoxins models. Several toxins have been utilized based on their mechanism of action for learning and memory impairment such as scopolamine, streptozotocin, quinolinic acid, and domoic acid. Further, some toxins like 6-hydroxy dopamine (6-OHDA), 1-methyl-4-phenyl-1,2,3,6-tetrahydropyridine (MPTP) and amyloid-β are known to cause specific learning and memory impairment which imitate the disease pathology of Parkinson’s disease dementia and Alzheimer’s disease dementia. Apart from these toxins, several other toxins come under a miscellaneous category like an environmental pollutant, snake venoms, botulinum, and lipopolysaccharide. This review will focus on the various classes of neurotoxin models for learning and memory impairment with their specific mechanism of action that could assist the process of drug discovery and development for dementia and cognitive disorders.

## 1. Introduction

Memory is the process that glues and holds our mental life together. Without memory, both our unconscious and conscious life would be like a disseminated and entangled mesh of unprocessed thoughts. We cannot perform our daily chores, and our life would become much more difficult to manage. Unstable memory can influence our cognitive potential and thus our quality of life at all stages of life. Premature ailments of learning and memory hamper the normal development of children while the unavoidable weakening of memory with time frustrates and irritates the natural aging [1]. During the last couple of years, neurobiological studies of the brain, has accomplished a common theoretical scaffold that expands from molecular and cell biology, on the one hand, to psychology and brain system biology, on the other [1]. The molecular and cellular foundation of learning and memory is an issue that has captivated neuroscientists for decades. The absolute intricacy of how we construe, remember, and forget our incidents seem impossible to understand at the cellular and molecular level. Through the use of many different learning and memory paradigms in different model organisms, we are beginning to have a basic understanding of the molecular changes that allow neurons to create and store memories [2,3]. Learning is the incidence-reliant attainment of skills and knowledge, whereas memory is the preservation and retrieval of events or facts composed of experiences [4]. Memory, as calculated by modifications in an animal’s behavior sometime after learning, mirrors various processes including acquisition, consolidation, retention, retrieval and performance. Molecular mechanisms of memory have focused mainly on the roadways that underlie acquisition. This emphasis is due, in large part, to the success of in vitro models of learning, including forms of synaptic plasticity such as long-term potentiation (LTP) [5]. Dementia can be defined as cognitive impairment in more than one cognitive area described by the loss of intellectual ability of sufficient severity to interfere either with occupational functioning, usual social activities or relationship of a person in the absence of gross clouding of consciousness or with motor involvement [6]. A study document from demographics of aging and memory [7], anticipated that in America the number of people with dementia was 4.5 million and by 2050 it might increase to 114 million [8,9]. Cognitive regions concerned in dementia includes: motor (apraxia), language (aphasia), executive functions (abstract reasoning, judgment and planning) and agnosia (failure in recognition) [10]. There are several types of dementias [11,12] including dementia of Alzheimer’s disease (AD), Huntington’s disease (HD) and Parkinson’s disease (PD), dementia with Lewy body, vascular dementia, frontotemporal dementia, Creutzfeldt-Jakob disease and Wernicke-Korsakoff syndrome. Our aging society has to deal with a significant rise in the incidence of age-related neurodegenerative diseases [12].

Hence, development of suitable animal models is essential to the drug discovery and development process so that new molecules are obtained that helps to overcome dementia and other memory disorders [13]. In addition, appropriate animal models of neurodegenerative conditions are precious to understand the pathophysiology of dementia and development of new therapeutics [14,15]. Due to the prevalence and poor prognosis of the disease related to memory, there is a high precedence for research to develop an animal model of dementia [16]. Development of animal models are demanding as there is no single animal model that can explicate all the biochemical, histopathological cognitive, and behavioral abnormalities [17]. An ultimate animal model should imitate the human disease and replicate complexities of human behavior in rodents. So far, various animals like monkeys, aged rhesus, rodents, worms and flies have been used to develop animal models of dementia. Rodent have an upper hand in developing good models since mice cannot develop plaques and tangles normally; whereas mice can be induced to develop plaques and tangles in specific brain regions [18]. Models involving primates take a longer time to develop and are expensive to maintain. Worm and fly brains are hugely different from human brains, and it is hard to develop dementia related disorders in them [16]. Various toxins (scopolamine, colchicines, streptozotocin, heavy metals, etc.) have also been used to develop animal models of dementia. Nonetheless no disease progression, high mortality and time consuming are the major disadvantage of these models [13]. On the other hand, the advantage of these toxin models is that they have been used to learn many therapeutic agents clinically in use for a variety of cognitive dysfunctions and dementia, thereby facilitating them to closely simulate with the pathophysiology analogous to humans. Recently more and more studies are underway to develop an ideal animal model that is inexpensive, non-invasive and mimics the pathogenesis of the memory disorders. In this review, as well as in Table 1, we will discuss specific mechanisms underlying the toxin-induced cognitive impairment in various experimental models. Based on their target toxicity of these toxins we would also discuss how these models can be used to investigate potential nootropic drugs in various memory disorders.

## 2. Toxin-Induced Experimental Models of Memory Impairment

### 2.1. Scopolamine-Induced Memory Impairment

The significance of cholinergic action in the brain for learning and memory functions was first recognized more than three decades ago, when comparatively low doses of certain muscarinic acetylcholine receptor antagonists (e.g., the belladonna alkaloids scopolamine and atropine) were established to induce temporary cognitive deficits in young human volunteers that resembled those seen in elderly (unmedicated) subjects [44]. It is well accepted that cholinergic system plays a vital role in the memory function [45,46]. Reduction in the function of the central cholinergic system may stimulate aspects of dementia like a failure of memory and puzzlement as seen in AD [47,48]. It has been recommended that the failure of cholinergic neurons typically occur in brain areas connected with memory and learning, such as the cortex, hippocampus, and nucleus basalis of Meynert [49]. Therefore calculating the number and strength of cholinergic neurons by Nissl staining in a variety of animal models indicative of dementia could have enormous significance to the current research in this field. AD patients demonstrate significant impairment of central cholinergic functions and loss of cortical cholinergic neurons [50]. Obstruction of central cholinergic function has been confirmed to stimulate memory loss [51,52].

Scopolamine, a well-recognized anticholinergic drug, is commonly used as a standard drug for the experimental purpose to induce cognitive deficits in animals. Administration of scopolamine produces deficits on tests of visual recognition memory, visuospatial praxis, verbal recall, visuospatial recall, psychomotor speed and visuoperceptual function [53]. It has been accounted that scopolamine nonselectively blocks the binding sites of acetylcholine (ACh) muscarinic receptors in the cerebral cortex and consequences in disproportionate release of ACh which destroys the hippocampus nerves and causes impairment in learning and memory in a dose-dependent manner in mice [54]. However, it seems that the central effects of scopolamine might be due to the blockage of M1 and (probably) M5 receptors because of their specific distribution in brain [55]. Recently it is been found that there is a possible involvement of *N*-methyl-d-aspartate (NMDA) receptor mechanisms of dorsal hippocampus and/or septum in scopolamine-induced memory impairment [56]. Very recently, hippocampal chromatin-modifying enzymes namely, histone deacetylase-2 and DNA methyl tranferace-1 are observed to be imperative for scopolamine-induced synaptic plasticity gene expression and following the decline in memory consolidation [57]. Infusions of scopolamine into the hippocampus blocks LTP [58] and impairs spatial encoding [59], and infusion into the medial septum impairs spatial learning and reduces ACh release in the hippocampus [60]. Infusions of scopolamine into region CA3 cause selective impairments of encoding but not retrieval in the Hebb-Williams maze [61]. Scopolamine was observed to cause spatial learning and memory deficits that involved stimulation of glycogen synthase kinase-3 beta (GSK-3β) and malfunctioning of spine formation/maturation and dendrite arborization associated with changes in CREB, Homer1, and α-amino-3-hydroxy-5-methyl-4-isoxazole propionic acid receptor (AMPA-R) [46]. Systemic administration of scopolamine elicits clear performance deficits in the radial maze [62]. Apart from the effects on acquisition, scopolamine particularly interrupts performance when hold up procedures were employed, which indicate that scopolamine exclusively impairs (spatial) working memory [55]. Systemic administration of scopolamine to animals subjected to Morris water maze has been documented to be more efficient in interrupting acquisition as compared to impairing retention [63]. Summing up, biological effects of scopolamine on tasks evaluating learning and memory were seen at higher (0.03 mg/kg) systemic doses. It seems that scopolamine is mainly successful in damaging acquisition/learning, short-term and working memory [55]. Scopolamine-mediated memory impairment is one of the most extensively used models since complex surgical events are not obligatory. The use of scopolamine in humans might be a useful tool to explore the role of acetylcholine in cognition. On the other hand side, since cholinergic neurons (or their projections) and, muscarinic receptors are found in almost each region of the CNS. Consequently the interruption of memory-related task performance by scopolamine could take place from a multitude of pharmacological actions [55]. However, such effects could also give rise to unpredictable results that have frequently been seen across several types of memory-related tasks [64].

### 2.2. MPTP-Induced Memory Impairment

Dopamine (DA) is a chief modulator of particular synaptic changes seen at certain stages of synaptic plasticity and learning and memory [65]. Additionally, DA serves to expand or contract the width of information held in working memory buffers in networks of the prefrontal cortex [66]. Substantia nigra pars compacta (SNpc) is an indispensable section of a memory system which is not dependent of the hippocampal memory system [67]. MPTP (1-methyl-4-phenyl-1,2,3,6-tetrahydropyridine) is a well-identified neurotoxin that destroys dopaminergic neurons [68] and has been used since decades to study models of PD. Traditionally, PD had been distinguished with specific hallmark motor symptoms. However, non-motor aspects such as cognitive deficits and dementia [69] caused by the demise of non-dopaminergic neuronal systems [70] are more and more recognized as part of PD [71]. Some of the earlier reports recommended that intra-nigral administration MPTP can produce a partial lesion in the SNpc [72], specific cognitive deficits (spatial working memory) [73,74] which are not dependent on motor deficits [75]. This protocol served as a model for early PD amnesia [76]. More recently, the model has been further modified to produce animals that consist of a motor and cognitive deficits of PD [77]. To construct an animal model of memory deficits observed in PD is a challenge, since this disease manifests motor impairment and, in learning and memory tasks, memory is articulated through a motor response.

The immunity of rats to a complete lesion induced with the MPTP presented us with the ideal conditions for such a model of PD. Intranigral injection of MPTP causes loss of DA in the dorsal striatum and prefrontal cortex, without altering the dopamine levels in the ventral striatum, hippocampus, or amygdala [78]. Unilateral delivery of MPTP to SNpc exhibits a decrease of the rearing behavior during an exploratory session to an open which is not observed in the bilateral model [72]. Therefore, bilateral MPTP-intoxicated rats in SNpc are specifically suitable for studies of learning and memory disabilities related to the early phase of PD [79]. In 1982, MPTP was discovered by coincidence in a synthesis process gone wrong, and, although it might have caused some chaos in certain circles, today it signifies the most important and most commonly used parkinsonian toxin applied in animal models [80]. It has frequently been confirmed that MPTP is certainly the gold standard for toxin-based animal models of PD among PD researchers for reproducing almost all of these hallmark features [80]. MPTP is extremely lipophilic molecule and after systemic administration, it quickly crosses the BBB. Once in the brain, MPTP enters astrocytes and is metabolized into its active metabolite, MPP^+^, by monoamine oxidase-B (MAO-B). Recent reports explain that once free from the astrocytes into the extracellular space via the OCT-3 transporter [81], MPP^+^ is absorbed into the neuron by the dopamine transporter (DAT) and can be stored in vesicles via uptake by the vesicular monoamine transporter (VMAT2) [82]. As a result, mice lacking the DAT have immunity against MPTP toxicity [83]. Once inside the neuron, MPP^+^ inhibits complex-I of the mitochondrial electron transport chain, causing oxidative stress and a decrease in ATP generation. Accumulation of MPP^+^ into vesicles can decrease its toxicity [84]. Furthermore, MPP^+^ stored in vesicles is considered to push DA out into the intercellular space where it can be metabolized into some toxic metabolites compounds, including, DOPAL and where it is can be subjected to superoxide radical (5-cysteinyl-DA) and hydroxyl radical [85] attack. MPTP-mediated degeneration of dopaminergic neurons and impairments in object recognition in rats are linked to neuroinflammation in the hippocampus [86]. In addition, decreased CaMK-II activity in the hippocampus is related to deficits in cognitive function in MPTP-treated mice [87]. The majority of the cognitive impairments exposed in MPTP-treated monkeys not only simulate those observed in PD patients but also mimic those found in patients having damaged frontal lobe [88]. Variable delayed response task, object detour retrieval task, and attentional set-shifting test are significant parameters to monitor the cognitive deficits in primates [88]. However, MPTP-exposed mice have not been specifically evaluated for cognitive dysfunction [89]. Reports that have evaluated features of cognition in rodent genetic PD models (primarily mouse models) have accounted an array of cognitive impairment, including diminished spontaneous alternation in a Y-maze [90] or T-maze [91], disrupted learning and retention in the Morris water maze [92], and impairments in operant reversal learning, object-place recognition, and novel object recognition [90]. Apart from some inconsistencies in the various reports, most likely due to the differences in the animal strain employed and in the protocol adopted, these animal models have been able to reproduce the cognitive decline observed in both early and late parkinsonian conditions [88].

### 2.3. 6-Hydroxy Dopamine-Induced Memory Impairment

The most common animal model used in the preclinical research of PD is based on the use of 6-hydroxydopamine (6-OHDA) [93]. Since 6-OHDA cannot cross the BBB, this neurotoxin needs to be administered directly into the target brain region [94]. Under normal physiological conditions 6-OHDA undergoes a rapid non-enzymatic auto-oxidation that produces toxic species such as hydrogen peroxide, superoxide radicals, quinones, and hydroxyl radicals [95]. There is general agreement that the toxic effect of 6-OHDA is due to the oxidative stress induced by the generation of ROS [96,97]. It is also supposed that 6-OHDA kills the dopaminergic neurons in medial forebrain bundle (MFB) by necrosis. However, mechanisms of progressive dopaminergic neuronal death induced by the striatal injection of 6-OHDA are not explored [98]. Regardless of this debate, the c-Jun N-terminal kinase (JNK) signaling pathway has been revealed to be involved in dopaminergic cell death in both the MFB and in the striatal models [99]. Lately, it has been established that apoptosis signal-regulating kinase 1 (ASK1) stimulates the JNK/c-jun pathway in 6-OHDA-mediated cell death [100]. It has been confirmed that cell loss both in the striatal [101] and the MFB models [102] follows microglial activation in the SNpc and may also account for the delayed phase of neurodegeneration [103]. Newly it has been revealed that unilateral injection of 6-OHDA in MFB, disseminates a cytoplasmic and mitochondrial protein known as Hsp60 by degenerated neurons to trigger microglial activation in the rat PD model [104]. Unilateral 6-OHDA intoxication into the MFB rigorously disrupts mitochondrial function within the lesioned SNpc [105].

There are various methods to inject 6-OHDA including the SNpc, the MFB, or the striatum. Direct administration of 6-OHDA into the SNpc or the MFB causes a quick and enormous degeneration of dopaminergic cell bodies that consequently involves the nigrostriatal pathway according to an anterograde progression. On the other hand, injection of 6-OHDA into the striatum initially affects the dopaminergic terminals and thus disrupts the striatonigral pathway and the projecting regions according to a retrograde succession [106]. For cognitive studies, bilateral injection of low concentration of 6-OHDA is preferred as it produces a balanced loss of dopaminergic neuron and mimics early stage of PD [107]. The use of PD rodent models achieved via injecting 6-OHDA in brain signifies a manageable, reproducible, and convincing tool for investigating cognitive symptoms such as memory impairment, executive dysfunction, and visuospatial deficits [108]. Bilateral exposure of 6-OHDA into the ventral and dorsal subiculum and adjacent CA1 hippocampus area [109] specifically damages the test performance in the spatial, but not in the cued, version of Morris water maze. Fascinatingly, a bilateral intoxication into rats’ MFB by 6-OHDA can damage the performance of the animals throughout the learning and testing in both versions of Morris water maze [110] as well as damages spatial memory examined in open field hole board test [111]. Bilateral administration of 6-OHDA in the mouse striatum particularly damages the performance in the long-term, but not in the short-term novel object recognition test [112]. Conversely, injections of 6-OHDA in the mouse striatum damages both the short and the long-term spontaneous object recognition [113]. One disadvantage of this model is, since, 6-OHDA has structure similarity to DA, it is taken up by catecholaminergic nerve terminals at the side of injection. Thus, to specifically target dopaminergic neurons, 6-OHDA has to be dosed together with inhibitors of noradrenaline and serotonin transporters [88].

### 2.4. Amyloid Beta-Peptide-Induced Memory Impairment

It is very well known fact that formation of amyloid-beta (Aβ) plaques are distinct characteristic features of AD and administration of Aβ peptide has been renowned for inducing memory loss [114]. Aβ is a 4 kDa polypeptide obtained by the proteolytic breakage of the Aβ precursor protein (APP) [115]. The main form of Aβ consists of 40 amino-acid residues. Although long Aβ (carboxy-terminally extended species) containing 42 or 43 residues are also produced [115]. These long forms of Aβ aggregate more efficiently as compared to the 40-amino-acid peptide. It is generally supposed that aggregated Aβ is accountable for progressive nature of disease, as the unregulated buildup of aggregates is toxic to the brain [116]. More recently an updated version of this theory serves a pivotal role in the pathogenesis of AD to soluble Aβ oligomers, which can quickly block LPT, and therefore cause memory deficits [117]. There are several biochemical pathways accountable for Aβ-mediated memory impairment. However, it has been tricky to conclude which of these pathways are the most significant in the onset of AD. Aβ-peptide-mediated inhibition of choline acetyltransferase (ChAT) to induce dysfunction of cholinergic neurons is one of the hallmark mechanisms of Aβ-peptide to induce AD [118]. Aβ augments hyperphosphorylation of tau and stimulates the development of neurofibrillary tangles in various cellular and animal models [119,120,121]. This insinuates us that Aβ might elicit its neurotoxic effects via hyperphosphorylation of tau and subsequent neurofibrillary tangle formation [122]. One more well-examined toxic effect of Aβ accumulation is disrupted calcium homeostasis. A soluble form of Aβ oligomers have been revealed to modify calcium regulation by altering ion channels, including potassium channels, nicotinic, NMDA receptors and voltage-gated calcium channels, and also by forming its calcium-conducting pores, thereby enhancing levels of cytosolic calcium [123,124]. Oligomeric Aβ has also been revealed to encourage the formation of ROS in primary cortical and hippocampal neurons [125], which has been connected with inflammation and neurodegeneration. Aβ may perhaps indirectly induce neurotoxicity via the stimulation of proinflammatory responses [126], caspase-8 [127] and ROS [128] which might have a role in restraining the formation of hippocampal memory [129]. Nonetheless, the majority of the direct effect of Aβ in on synaptic degeneration through its action on precise signaling cascades.

Aβ blocks LTP particularly interfering with CREB and calcium/calmodulin-dependent protein kinase II (CaMK-II) signaling pathways [130]. Aβ treatment inhibits the PI3K/AKT and Ras/ERK signaling pathways [131], which are downstream of BDNF/TrkB but are also indispensable for the phosphorylation of CREB and ensuing the expression of BDNF. Aβ-42 also has been reported to exhibits its toxicity by activating the MAPK cascade through hippocampal α7 nicotinic ACh receptors in cellular and animal model of AD [132]. Aβ also is found to inhibit the distribution of RACK1 (receptor for activated C-kinase 1), in cortical neurons and in this manner affect the working memory [133]. Neurodegeneration mediated by Aβ-oligomer is associated with cytosolic phospholipase A2 (cPLA2) and plays a vital role in the early synapse loss and cognitive impairment [134]. Features of AD can be simulated by intracerebral or intra-cerebroventricular (ICV) infusion of Aβ peptides in the rodent brain [135]. Inside the published literature there is an extensive disparity in the documented behavioral and neuropathological effects of Aβ infusion. These discrepancies may be due in part to deviation in methodologies; the site of infusion, concentration and the state of aggregation, the species of peptide infused (e.g., Aβ1-40, Aβ1-42 or Aβ25-35), the peptide preparation, the duration of infusion, the time interval between Aβ administration and behavioral testing, and even the solvent used for peptides [135]. Aβ oligomer can be administered acutely, using a single stereotactic injection [136], or chronically, using injections through an implanted cannula [137]. To better simulate the progressive nature of AD, chronic and continuous administration of Aβ peptide can be achieved by linking an implanted cannula to an osmotic mini-pump [138] or by using a micro-infusion pump [139], or with microdialysis [140]. It has been established that continuous infusion or acute injection of Aβ into the brain leads to brain dysfunction followed by neurodegeneration and impairment of learning and memory very similar to that seen in AD [141]. Infusion of Aβ into the 3rd ventricle of the rat brain for 14 days leads to Aβ accumulation in various areas e.g., hippocampus and cerebral cortex [142]. This model is highly specific for screening of drugs used in AD.

### 2.5. Streptozotocin-Induced Memory Impairment

Streptozotocin (STZ), chemically is a glucosamine nitrosourea obtained from a soil microbe Streptomyces achromogenes [143]. STZ acts as an alkylating agent which simulates some features of anticancer agents (nitrosoureas) which is used in pancreatic carcinoma. STZ has been extensively investigated for its potential to induce diabetes in animals [144]. The ICV STZ animal model was introduced by Lannert and Hoyer in 1998 [145]. ICV injection of STZ at a sub-diabetogenic dose (3 mg/kg), twice at an interval of 48 h in rodents elicits a progressive loss of memory seen very analogous to that of AD [146]. ICV STZ-mediated memory deficits are not dependent on its hyperglycemic effect [147]. Modifications in LTP-like appearance of synaptic plasticity in other cerebral structures and in the hippocampus are accountable for learning and memory deficits by STZ [148]. However recently, ICV injection of STZ has been recognized to generate neuronal damage by oxidative stress via the generation of ROS and reactive nitrogen species [149], increase in the levels of malondialdehyde, accumulation of Aβ in the brain, hyperphosphorylation of tau protein, as well as negative regulation of the genes linked to insulin signaling such as IGF-1 receptors [150]. On the other hand, hippocampal insulin receptor system may also be playing an imperative role in the regulation of memory functions. However, only insulin receptor/insulin receptor system-1/Akt pathway in CA3 region of the hippocampus is responsible for STZ induced memory deficit [151]. Recently, liver-X receptors are also implicated in the pathophysiology of dementia induced by STZ [152]. Data have established that ICV injection of STZ causes a reduction in activities of glycolytic key enzymes in the hippocampus and cerebral cortex, which in turn causes a decrease in the levels of ATP and creatine phosphate [153]. As an outcome of damaged energy metabolism in the brain, there is a reduction in synthesis of acetyl coenzyme-A and disordered cholinergic transmission. Specifically, decreased the activity of ChAT in the hippocampus of STZ exposed rats has constantly been reported [154].

Furthermore, augmented activity of acetylcholinesterase (AChE) has been found in the brain of STZ exposed rats which might be an outcome of increased degradation of ACh, thus improving the deficits in ACh produced by fall in ChAT expression in these animals [155]. Data suggest that changes in the total and phosphorylated GSK-3α/β have also been established after icv injection of STZ, which have been recommended to be linked to the formation of Aβ peptide-like aggregates [52]. Moreover, STZ injection has also been confirmed to produce brain atrophy, mainly due to oligodendroglial and neuronal cell loss induced by apoptosis, mitochondrial dysfunction, neuroinflammation and oxidative stress. The main benefit of this model is that it strongly mimics some pathology of human sporadic AD [156]. The drawback of this model is due to high mortality rate and large numbers of animals are required [157]. However, the efficiency of most of the therapeutic plan investigated in STZ-ICV model has also been tested in AD patients, and similar results have been obtained in many of the clinical trials [158].

### 2.6. Quinolinic Acid-Induced Memory Impairment

Quinolinic acid (QA), is a well known NMDA receptor agonist frequently used to induce HD in various experimental models [159]. QA administration produces many neurochemical and histopathological trends of HD neuropathology and also causes memory deficits [160]. QA, a neuroactive metabolite of the kynurenine pathway, usually exists in nanomolar concentrations in human brain and cerebrospinal fluid (CSF) and is frequently responsible for the pathogenesis of a range of human neurological diseases. Apart from being a NMDA receptor agonist, it has a high in vivo potency as an excitotoxin [161]. Even though QA has an uptake system, its neuronal metabolizing enzymes are quickly saturated, and the remaining extracellular QA can carry on activating the NMDA receptor. Nevertheless, its toxicity cannot be completely clarified by its stimulation of NMDA receptors. It is expected that supplementary mechanisms may also be implicated in its toxicity [161]. Particularly, neurons within the hippocampus, neocortex and striatum are sensitive to QA but cerebellar, and spinal cord neurons are less sensitive. These variations in regional sensitivity most probably relate to variations in the configuration of NMDA receptors [162,163]. Recently it is been demonstrated that by using a combination of three NMDA receptor antagonists, the toxicity of QA towards motor neurons could be fully reversed [164]. QA has been observed to augment the release of glutamate by neurons, slow down its uptake by astrocytes and restrain the activity of glutamine synthetase in astroglial cells leading to disproportionate glutamate concentrations and neurotoxicity [165]. Another main mechanism of QA neurotoxicity is via lipid peroxidation [166]. Few studies have suggested that QA forms a complex with iron (Fe), and electron transfer from this complex to oxygen causes the formation of reactive oxygen species (ROS) which then carries out lipid peroxidation [167,168]. QA-Fe(II) complexes display significant pro-oxidant characteristics that could have implications for QA neurotoxicity [169]. This may partially explain the beneficial effect of antioxidants on excitotoxic insults [170]. QA can augment its toxicity and that of other excitotoxins (e.g., glutamate and NMDA) on energy depletion [171].

It is also possible that QA toxicity is synergistic or additive with other immune-system-derived toxins, such as glutamate, glycine and NMDA which either modulate or act via the NMDA receptors [172]. Few experiments have demonstrated that QA is capable of distorting the integrity of the blood-brain barrier (BBB) [173,174]. Fascinatingly, some areas of the striatum and in the hippocampus were extra sensitive to QA excitotoxicity than others [174]. QA activates neuronal nitric oxide synthase (NOS) and inducible nitric oxide synthase (iNOS) activity in human neurons, rodent, and astrocytes, thus leading to a bigger NO production [175]. Two recent reports have demonstrated that QA can augment the phosphorylation of cellular structural proteins causing to destabilization of the cytoskeleton [176]. Intrastriatal injection of QA in the brain of young rats results in a considerable rise in the phosphorylation of low molecular weight neurofilament subunits in glial fibrillary acidic protein (GFAP) in astrocytes and neurons. At pathologic concentrations, QA mediates the expression of several proinflammatory chemokines and cytokines such as IL-8, monocyte chemotactic protein-1, and IL-1β in primary human astrocytes [177]. Recently Guillemin and associated have demonstrated that the expression patterns of cathepsin D, a lysosomal aspartic protease, are amplified after intoxication with QA. Hence, a discrepancy in autophagy is expected to signify a new and considerable mechanism for QA toxicity in both neuronal cells and human glia [178]. Chronic injection of QA (20 mM) in the rat striatum induces spatial learning deficits in a radial arm water maze task [179]. Striatal lesions produced by intrastriatal injection of QA impaired hidden-platform Morris water maze acquisition training in rats [180]. Escape latency throughout the hidden-platform acquisition training was greater than before in rats with bilateral striatal lesions after intrastriatal injections of QA [181]. Lesion of QA to the rat entorhinal cortex pars medialis induces specific amnesia in allocentric, but not in egocentric working memory [182].

### 2.7. 192 IgG-Saporin-Induced Memory Impairment

192 IgG-saporin is the one of the first active anti-neuronal in vivo immunotoxin [183]. 192 IgG-saporin consists of the monoclonal antibody 192 IgG which is disulfide attached to saporin. Saporin has known as ribosome-inactivating protein (RIP) since it catalytically demolishes ribosomal RNA thus stopping protein system and stimulating cell death via apoptosis [184]. Physiologically, cholinergic neurons in the cholinergic basal forebrain (CBF) include p75-neurotrophin receptors which orchestrate the effects of rat nerve growth factor (NGF). NGF plays a role in the maintenance of function of the magnocellular CBF neurons, which have NGF receptors [185]. The antibody component of 192 IgG-saporin has aimed against low-affinity NGF receptor (p75^NGFFR^). Targeting p75 provides is a perfect way to lesion the CBF neurons specifically since in the adult rat basal forebrain; only the cholinergic neurons express p75. Cholinergic neurons have p75-NGFreceptors while neurons possessing other neurotransmitters in the region including the adjacent striatal cholinergic interneurons, do not express measurable levels of p75-NGFreceptors [183]. Systemic injection of 192 IgG-saporin causes it to bind to the surface of p75-containing neurons and then it is internalized by endocytosis. Once in the cytoplasm, the saporin part of 192 IgG-saporin escapes endosomes and enzymatically inactivates the large ribosomal subunit and thus inhibiting the protein synthesis and ultimately causing cell death [186].

Hawkes and colleagues found that specific loss of basal forebrain cholinergic neurons by 192 IgG-saporin is related with diminished phosphorylation of Ser^9^ GSK-3β [187]. Both direct injection and ICV administration of the 192 IgG-saporin into selective basal forebrain nuclei induce almost complete and specific lesions of cholinergic cells by sparing other neuronal systems in the basal forebrain [183]. 192 IgG-saporin ICV injections also result in loss of cholinergic markers such as ChAT activity, high-affinity choline transport (HAChT) and AChE positive fibers, in the target fields of the CBF [184]. Apart from CBF, icv injection of 192 IgG-saporin also affects purkinje cells of the cerebellum [188], since a subset ofthese cells starts expressing p75 receptors of NGF during development and continues untill adulthood [189]. High-dose icv administration of 192 IgG-saporin (4–10 mg) results in impairment in the acquisition of navigational learning in Morris water maze and retention memory deficits in passive avoidance test [190,191]. Icv intoxication of 192 IgG-saporin destroys spatial learning and working memory. Many experiments have reported deficient performances in radial arm maze [192,193] and in a water version of radial arm maze [194] following icv injections of 192 IgG-saporin. Conclusively, 192 IgG-saporin is the most efficient and specific tool for producing lesions of the rat CBF. Its advantage over previous techniques is in its capability to specifically kill cholinergic neurons of the basal forebrain and to spare other intermingled non-cholinergic neurons. Important drawbacks of 192 IgG-saporin include damaging some cerebellar purkinje neurons and sparing of the cholinergic innervations to amygdala [183].

### 2.8. Okadaic Acid-Induced Memory Impairment

Okadaic acid (OKA) is one of the chief polyether toxins obtained from marine microalgae which induces diarrhetic shellfish poisoning [195]. ICV administration of OKA-induced memory impairment in rat serves to be a useful test model to screen anti-dementia drugs [196]. With respect to the mechanism of action, OKA is a selective and potent antagonist of serine/threonine phosphatases 1 and 2A [197,198], affects short and long-term memory alteration in rats [199], stimulates hyper-phosphorylation of tau protein, and neuronal cell death in vitro [200] and in vivo [201]. OKA decreases basal synaptic transmission and inhibits the initiation of synaptic plasticity in the form of [202]. OKA also augments Ca^2+^ in hippocampal neuronal cell culture via ionotropic excitatory amino acid receptors leading to neuronal degeneration [203]. OKA stimulates generation of ROS in the hippocampus, lowers mitochondrial activity and mitochondrial membrane potential leading to mitochondrial dysfunction in rat brain [204]. OKA also activates expression of heat shock protein, neurodegeneration in rat hippocampus in vivo. Since OKA inhibits phosphatases, it also induces hyperphosphorylation of protein and thereby induces neuronal stress and subsequent neurodegeneration [196]. OKA-mediated cognitive-impairment in rats is associated with increased expression of proinflammatory cytokine TNF-α and IL-1β, iNOS and total nitrite in hippocampus and cortex [205]. Bilateral injection of OA in hippocampal produces spatial cognitive deficit by hippocampal gliosis (increased expression of GFAP), lowers GSH and an augments protein carbonylation and p38MAPK [206].

Numerous protein kinases, including mitogen-activated protein kinase (MAPK),GSK-3, and cyclin-dependent kinase 5 (Cdk5), have been documented to phosphorylate tau protein at a range of sites that are found in AD hyper-phosphorylated tau [207,208] whereas its dephosphorylation is mostly catalyzed by protein phosphatase (PP) 1, 2A, 2B and 5, with PP2A as the chief player [209,210]. Any discrepancy between tau dephosphorylation and phosphorylation is decisive for AD tauopathy [209]. Specific blockade of PP2A by OKA can produce an Alzheimer-like hyperphosphorylation and accumulation of tau both in vitro [211,212] and in vivo [213]. Memory impairment induced by intra-hippocampal injection of OKA has been associated with significant neuropathological changes including a paired helical filament-like phosphorylation of tau protein, formation of Aβ containing plaque-like structures and hippocampal neurodegeneration [206]. OKA is an exceptionally useful tool for investigating the cellular processes that are modulated by reversible phosphorylation of proteins as cell division, signal transduction, and memory [214]. None of the drugs are available, which acts by blocking tau hyperphosphorylation. Hence it can be suggested that OKA will be a promising substitute tool to explore a therapeutic approach for AD pathology [195].

### 2.9. Domoic Acid-Induced Memory Impairment

Domoic acid, a tricarboxylic amino acid is a kainate receptor agonist that is structurally related to kainic acid (KA) and glutamic acid. Microinjections of domoic acid, a presumed shellfish toxin, into the hippocampal regions of rats induce degeneration of CA1 and CA3 pyramidal cells and dentate gyrus granule cells [215]. Neurodegeneration induced by domoic acid results in rigorous deficit in short-term memory that is mediated by Ca^2+^ overload and inhibition of Ca^2+^ and calmodulin-stimulated adenylate cyclase [216]. Domoic acid stimulates the (AMPA)/KA receptor AMPA/KA-R, which produces increased levels of intracellular Ca^2+^ which, in turn, leads to the release of glutamate that consequently activates the NMDA-R [217,218]. Direct and indirect stimulation of AMPA/KA-Rs and NMDA-Rs can cause apoptotic and necrotic neuronal cell death [219,220]. The method of neuronal cell death, necrotic or apoptotic, in pure neuronal cultures seems to depend on the concentrations of domoic acid. Since high concentration (10 μM) mediates necrosis also through glutamate release and secondary activation of NMDA-R and a low concentration (0.1 μM) mediates apoptosis mostly via AMPA/KA-R [221]. Furthermore, the exposure time has also been recognized as a central feature that might boost the domoic acid-induced toxicity [222]. Furthermore, a few studies have suggested involvement of glial cells that could enhance the domoic acid-mediated neurotoxicity [223].

Reports by Petrie and associates have documented that rats treated with domoate display long-lasting anterograde amnesia for the spatial task in the Morris water task [224]. In comparison to saline controls, animals intoxicated with domoic acid (2.0 mg/kg) exhibited considerable impairment on the acquisition of the place task in the Morris water maze. Surveillance of swim paths chosen by mice searching for the underwater platform exposed a failure on the part of the domoic acid-treated mice to select the suitable problem-solving strategies [225]. Intraparenteal (i.p.) injections of domoic acid to mice or rats (4 mg/kg) elicited lesions in the thalamus, olfactory cortex amygdala, and cortical mantle [226]. However, the most frequent site of neuronal damage is in the hippocampal structure and septal nuclei [226].The hippocampus and its projections are also injured in humans and primates by intoxication with domoic acid [227,228]. A single i.p. injection of domoic acid (2.0 mg/kg) induces a remarkable impairment on the place version of the Morris water maze in DBA strain mice. As compared with saline treated controls, domoic acid exposed mice had notably higher escape latencies for all acquisition days and all but the first day of reversal [225]. Direct microinjections of domoic acid into the hippocampus of rats also produces considerable deficits in the spatial but not the non-spatial components of the Morris task [224]. Domoic acid-exposed rats have deficits in the spatial acquisition and during a 30 s probe trial. Rats exposed to a single i.p. dose (1.5, 3.0, but not 0.75 mg/kg) have also been documented to have impaired performance in the Morris water maze [229]. Humans exposed to domoic acid have an inability to form new memories adequately [228]. As an experimental tool in neuroscience and neurotoxicology, domoic acid has an exclusive place as a gradually desensitizing AMPA/KA-R agonist that exhibits high potency and efficacy in a broad range of experimental systems [215].

### 2.10. Ethanol-Induced Memory Impairment

Exposure to ethanol at high doses have been documented to produce retrograde amnesia and disruption of encoding, storage, consolidation, retrieval potential and impair memory [230]. Data indicates that disruption of memory by ethanol is due to its effect on attention, sensory-motor function, emotion or motivation that impairs the efficiency of learning and encoding [231]. Ethanol might induce destructions of hippocampus-dependent learning and memory [232,233] and impair of the cholinergic neuronal system [234] by oxidative stress via the generation of ROS and lipid peroxidation [235]. Ethanol-induces presynaptic dysfunction in dorsal hippocampal glutamatergic neurons and thereby causes deficits in spatial memory [19]. The intoxication of infant mice to ethanol on a single postnatal day (P7) produces widespread apoptotic neurodegeneration in the developing brain, and consequent spatial learning and memory impairments that are extremely severe at P30, less severe if the investigation is performed at P75, and nominal in later adulthood [236]. Exposure to also spatial learning and memory, via activation of the κ-opioid receptor by increased glutamate overflow and activation of dynorphins and in the CA3 region [237]. In addition to this, effects of acute ethanol treatment are preferentially mediated through GABAergic systems [238], increased production of NO and NOS activity in brain regions which are linked with memory impairment such as hippocampus, amygdala and prefrontal cortex (PFC) [239].

Pathological doses of ethanol obstruct glutamatergic action by acting on AMPA, kainate and NMDA receptors and it also increases synaptic transmission of GABA in memory associated areas of the brain such as the hippocampus [240]. It has also been documented that ethanol enhances extracellular levels of adenosine that causes dysfunction of memory related various cellular cascades [241]. In a neonatal model of ethanol-mediated memory impairment, it is seen that pups born to alcohol-fed female rats illustrate deficits in acquisition in adulthood [242]. It has also been reported that acute (0.5–1 g/kg) [239] and chronic (15 *w*/*v*%, 2 g/kg, p.o. for 24 days) exposure to ethanol also displays deficits in memory [243]. The advantage of this model is that it does not necessitate any surgical procedures when used for evaluation of various memory enhancers. The drawback of this model is that the method is very long and time-consuming since pregnant female rats are used. Ethanol-mediated memory impairment is significantly related to state-dependency, which can also be affected by the quantity of its exposure [244].

### 2.11. Colchicine-Induced Memory Impairment

Colchicine is a potent cytotoxic agent which causes depolymerization and inhibition of microtubules by irreversibly binding to it. Microtubules are very important apparatus of the neuronal cytoskeleton and play a vital role in cell differentiation and growth, axonal and dendritic transport. It has been documented that central administration of colchicine produces memory deficits in rodents by cholinergic neurodegeneration and oxidative stress [245,246]. Colchicine inhibits axonal transport and augments neurofibrillary degeneration. Colchicine causes hippocampal lesions leading to learning and memory deficits, a decrease in ChAT, indicating that it could be used as an appropriate model for studying AD [247]. Colchicine can provoke neurotoxicity and memory impairments by the destruction of cholinergic pathways, loss of cholinergic neurons, and reducing cholinergic turnover mainly in the hippocampus area of the brain [248]. Memory impairments observed in colchicine is also due to fall in dopamine, nor-epinephrine and serotonin in the cerebral cortex, caudate nucleus and hippocampus [249].

Colchicine induces lipid peroxidation and protein carbonyls [250]. Colchicine is also reported to increase expression of cyclooxygenase-1 and 2 [251], generation of ROS [252]. Colchicine increases the glutamate/GABA ratio in the brain cortex [246] and stimulates hyperactivation of NMDA receptors which result in an increase in the influx of Ca^2+^, consequently leading to stimulation of Ca^2+^ dependent enzymes like phospholipases A2, proteases, xanthine oxidase, cyclooxygenases and protein kinases [6]. ICV administration of colchicine (7.5 μg in 10 μL) has been established to produce considerable memory deficits in rats [253] as well as in mice [254]. Noteworthy deficits of memory is marked after two weeks [255]. Intracerebral injection of colchicine at a dose of 3 μg/mice produces spatial memory impairment [256]. The major benefit of this model is that it simulates definite features of sporadic dementia of Alzheimer’s type (SDAT) in humans such as time-dependent alterations in behavioral, biochemical pattern and onset [250]. The drawback of this model is that it is time-consuming, necessitates the use of many animals due to high mortality rate. Decrease in appetite, and transient diarrhea, adipsia, and aphasia after 7–10 days of its administration are also some of the limitation of colchicine induced memory impairment [157].

### 2.12. Trimethyltin-Induced Memory Impairment

Trimethyltin (TMT) chloride is a potent organotin neurotoxicant that specifically induces neuronal death in hippocampal neurons of both animal and human limbic system [257,258]. In various animal experiments, administration of TMT induces cognitive deficits (learning impairment and memory loss) referred to severe hippocampal neuronal damage [259,260]. TMT is presently regarded as a valuable tool to attain an animal model of neurodegeneration linked with cognitive impairment and seizures [259,261]. Rats dosed with TMT show some or all of the subsequent behavioral deficits in radial-arm and water-maze acquisition, irritability, hypothermia, weight loss, tremor, seizures, tail mutilation. Furthermore, TMT-induced injury to the hippocampus, entorhinal cortex, and neocortical linked areas seem to be responsible for the behavioral deficits seen in TMT intoxicated rats [262]. In contrast to an acute lesion of the basal forebrain that demolishes the nucleus basalis, the neuropathological alterations produced by TMT continue for as long as 60 days after one single exposure. Therefore, the effectiveness of nootropic drugs projected to alleviate progressive behavioral impairment can be tested in this model [262]. Accidental humans exposed to TMT develop a syndrome distinguished by memory deficits, seizures, confusion, disorientation, and aggressiveness [263]. Animals, exposed to TMT induces neurotoxicity due to the preliminary oxidative stress [264,265]. Site-specific delivery of TMT-induced selective neuronal cell death and neuroinflammation that ultimately contribute to neurodegeneration [266].

Several experiments involving cells and animals have demonstrated that c-Jun N-terminal kinase (JNK) signaling, cyclooxygense-2, caspases-3/-8 and different proinflammatory cytokines are stimulated by TMT exposure and might be implicated in the pathological mechanism of TMT-mediated brain injury [267,268,269,270]. Mice intoxicated to TMT induces memory deficits, and neuronal cell loss, seizures, hyperactivity, especially in the hippocampal dentate gyrus (DG) [271,272]. Recently, some studies have indicated the phosphoinositol 3-kinase (PI3K)/Akt pathway to be a target for neuroprotection in TMT-induced injury to central nervous system (CNS) [265,273,274]. Thus, TMT-mediated neurotoxicity is considered as a valuable tool for the investigation of neurodegenerative diseases and hippocampal dysfunction, such as AD [266]. Recently, GSK-3 signaling pathway is seen to play an important role on in TMT-mediated hippocampal neurodegeneration in mice [275]. Thus, various new signaling molecules and pathways are discovered that are unraveling the mechanism of TMT-induced CNS toxicity. However, effects of TMT differ significantly with species. In addition, the efficacy and potency of TMT depends on its batches of manufacturing [262].

### 2.13. Ethylcholine Aziridinium-Induced Memory Impairment

Ethylcholine mustard aziridinium ion also denoted as (AF64A) is a highly neurotoxic derivative of choline that generates long-term deficits in presynaptic cholinergic output in rodents [276]. AF64A is identified to generate a selective decline in cholinergic presynaptic markers, such as high-affinity choline uptake, and ChAT activity without affecting monoaminergic systems [277,278,279]. AF64A targets the central events that control the synthesis of ACh. Physiologically, high-affinity choline transporters absorb back all the choline into the cholinergic nerve terminal (HAChT). Once inside, it is acetylated by ChAT to produce ACh. AF64A is a cytotoxic analog of choline that unites a choline-like structure (i.e., ethylcholine), that is accepted by the HAChT system, with a highly reactive cytotoxic aziridinium ring. Due to its analogous structure compared to choline, AF64A is taken into the terminal by the HAChT system and once inside the terminal, the extremely reactive aziridinium stimulates cholinergic hypofunction by damaging enzymes that use choline as a substrate such as AChE, ChAT, choline dehydrogenase, and choline kinase, by alkylating their catalytic sites [276] and thereby stimulate cell death. The overall mechanisms of neuronal demise are still under examination but they possibly involve the generation of oxidative stress and its impact on function of nucleic acid [276]. Icv administration of AF64A in mice considerably lowers ACh contents and ChAT activities in the hippocampus, striatum and cerebral cortex [277,278,280]. Since AF64A induces cholinergic hypofunction, it has been widely used to develop an animal model of dementia in AD [281]. The performance deficits induced by AF64A could be alleviated by treatment with cholinergic drugs such as arecoline and physostigmine [282]. AF64A intoxications cause impaired performance in learning and memory tasks with a corresponding decrease in ACh content in the hippocampus [283,284]. One recent study demonstrated that icv administration of AF64A at 6 nM impaired acquisition of Morris water maze task and performance in the probe test and histochemical examination after 20 days elicited clear cholinergic hypofunction [285]. Similarly, in another study, bilateral ICV injection of AF64A (2 nmol/2 uL) is used as an animal model of memory impairment and the neurodegeneration for AD in the hippocampus. AF64A administration notably decreases the neuron density in CA2, CA3, and dentate gyrus, augments the escape latency but diminishes retention time in Morris water maze [286]. AF64A also produces an extended time course of degeneration of cholinergic neurons which evolves over several weeks. Hence, AF64A provides a valuable model of cholinergic degeneration in which the cellular proceedings that contribute to cell death can be examined [276].

### 2.14. Ibotenic Acid-Induced Memory Impairment

Ibotenic acid (Ibo) was first discovered by Johnston et al. in 1968. Ibo is a heterocyclic amino acid obtained from mushrooms of the Amanita genus and structurally related to glutamate. Ibo is more potent neuro excitant in comparison to glutamate in Renshaw cells and spinal interneurons in cats [287]. Later, Schwarcz et al. in 1979 demonstrated that intracranial injections of Ibo induces degeneration of healthy neuronal population [288] and is also characterized impairments in ChAT activity and spatial learning and memory performance [289]. Injection of Ibo into hippocampal CA3 was found to mediate its toxicity via stimulation of NR2A/NR2A NMDA subunits that negatively influence cholinergic receptors causing memory impairment and deficits in locomotor behavior [290]. Ibo damages cholinergic neurons in the of nucleus basalis magnocellularis (NBM). Ibotenic acid is a NMDA receptor agonist which results in neuronal toxicity by overloading calcium into the neurons [253]. Ibo-mediated lesions of the medial septum increase activity of protein kinase C-associated with hippocampal membrane and decrease synthesis of Ach [291]. Ibo, have been recognized to be used to create fiber-sparing lesions restricted to the hippocampal region in monkeys [292,293]. Ibo specifically destroys cell bodies within the target region, sparing adjacent white matter [294]. Ibo damages recognition memory in rats [295] and five different groups of monkeys [296]. The combination of Ibo with Aβ (1–40) shows a noteworthy neuronal loss as compared to the individual toxicity shown by Ibo and Aβ peptide when used alone [297,298,299,300]. Ibo-mediated injury creates about 30% decrease activity of ChAT and injures wide regions of the ventral pallidum and substantia innominata [301]. Depending on the effects of Ibo on cholinergic and subsequent cognitive changes, intracerebral injection of Ibo [302,303] and lesion to NBM by Ibo [304] serves as a authenticated model of AD. In this model, unilateral injection of Ibo (10 μg/rat, dissolved in 5 μL of artificial cerebrospinal fluid) to NBM produces considerable memory loss [305]. In comparison to kainic acid, Ibo has the upper hand of being less toxic to the animals and of developing more distinct lesions, perhaps due to other fundamental biochemical differences and/or faster metabolism. Based on these special features, Ibo appears to symbolize a valuable new tool in the functional and morphological analysis of CNS [288].

### 2.15. Metals-Induced Memory Impairment

The range of experiments has displayed that metals like copper (Cu), chromium, cobalt, aluminum (Al), iron, and zinc (Zn) can boost the formation of ROS resulting in the development of dementia of AD and other types [306,307]. Metals such as lead (Pb), arsenic (As) and cadmium display their toxic effects through bonding to sulfhydryl groups of proteins and depletion of glutathione [308]. Recently toxicity of As has been found to induce endothelial dysfunction system and dementia in rats [309]. As negatively regulates the gene expression at the postsynaptic density in mouse cerebellum, as well as genes accountable for LTP and depression [310]. Recently, Pb has been revealed to dysregulate the activity of serine/threonine protein phosphatases in human neurons [311]. In addition, Pb has the potential to stimulate activation of microglia, which can further induce TNF-α, IL-1β, and iNOS. These proinflammatory factors have the ability to injure hippocampal neurons as well as produce deficits in LTP [312]. Zn has been revealed to stimulate dimerization of Aβ and is observed to be involved in the development and progression of AD [313,314]. It has also been verified that Cu leads to the generation of ROS which is a preliminary event in the development of AD [315]. Surplus levels of Al in drinking water have been reported to induce AD by interfering with Aβ metabolism as it interacts with insulin degrading enzymes [316,317]. It has been observed that exposure of Al leads to accumulation of tau and apoptosis that further causes neuronal dysfunction [318]. When Al bypasses gastrointestinal and BBB, it mounts up in hippocampal large pyramidal neurons and thus manifest symptoms of AD [319]. On the other hand, substantial facts have been submitted for an interaction of Al with ATP synthesis through oxidative phosphorylation. It has been lately established that exposure of astrocytic cells with Al results in mitochondrial dysfunctional and a loss of energy synthesis [320]. Additionally, it has been accounted that Al was able to block degradation of plasmin of the Aβ peptide [321] and is also reported to impair hippocampal LTP in rat [322] by inhibiting cAMP-PKA-CREB signaling and changing the neuronal and synaptic ultrastructure in the hippocampus [323].

## 3. Conclusions

Considerable development has been made during the last decades in understanding the pathophysiology of memory disorders. Toxins, either natural or synthetic have been important tools for investigating the molecular, biochemical, and morphological correlates of brain function. Toxins that interrupt precise neuronal populations, axoplasmic transport processes, ion channels, or neurotransmitter systems have helped to illustrate the primary neurobiology of brain cells and the principles of synaptic transmission. Neurotoxins have been used for decades to (1) explore the steps involved in the synthesis, storage, and release of neurotransmitters; (2) To establish how a transmitter networks with ion channels and specific receptors; (3) To explore the nature and extent of neural reorganization following its injury and to investigate the plasticity of a brain system; (4) To inspect the difference between altered behavior and neurotransmitter dynamics; and finally (5) build animal models of neurological disorders such as AD, PD, HD and temporal lobe epilepsy. None of the model’s accessible precisely simulate the cognitive, behavioural, histopathological and biochemical abnormalities displayed by in neurological disorders characterized by cognitive impairment. However, most of these features can be reproduced by using specific toxins. On the other hand, as a secondary option, many researchers are using the transgenic animal model as they offer to mimic the disease pathology closely. However, the developing cost and maintenance of these models is high and thereby limits its use on a larger scale.

## Figures and Tables

**Table 1:** Miscellaneous toxin-induced models for memory impairment.

Toxin	Mechanism of Action	Experimental Model	Type of Memory Impairment	References
Diazepam	Suppression of LTP, reinforcement of GABAergic transmission	Morris water maze task and modified elevated plus-maze task	Anterograde amnesia, spatial memory deficits	[19,20]
Delta9-tetrahydrocannabinol and WIN55, 212-2	Reinforcement of GABAergic transmission, molecular interaction between CB1R and 5-HT2AR	Step through test, novel object recognition test	Spatial memory, working memory, verbal learning deficits	[21,22]
Glucocorticoids	Site-preferential downregulation of hippocampal GC receptors, involution of the dendritic processes of hippocampal neurons, inhibition of long-term potentiation	Inhibitory avoidance task, Morris water maze task	Long-term memory impairment, Spatial memory deficit	[23,24,25]
Galactose	Induces oxidative stress which triggers memory impairment	Y-maze task	Spatial learning and memory impairment	[26]
Diisononyl phthalate	Oxidative stress, inflammatory responses, apoptosis, and hippocampus pathological alterations	Morris water maze task	Spatial learning deficit	[27]
Harmaline	Involvement of serotonergic system of the dorsal hippocampus, Involvement of CA1 dopaminergic mechanism, interference with the GABAergic systems	Initial learning test, Retrieval test	Spatial learning and memory deficits	[28,29,30]
Homocysteine	Accumulation amyloid and tau protein, activation of NMDA receptors	Morris water maze task	Impairment of short- and long-term memories	[31,32]
Melamine	Impairments of hippocampal long-term depression and cholinergic system, oxidative stress in hippocampus	Morris water maze task	Spatial cognitive deficits	[33,34]
Sodium azide	Inhibits mitochondrial respiratory chain, produces free radicals, diminishes aerobic energy metabolism and causes excitotoxic damage, decreases cholinergic input to the hippocampus	Morris water maze task, step-through passive avoidance	Spatial learning and memory deficits	[35]
Lipopolysaccharide	Oxidative and proinflammatory stress	Radial arm-maze task, Y-maze task	Spatial memory deficits	[36]
3,3′-Iminodipropionitrile	Morphometric changes in the hippocampus	Passive avoidance task, Y-maze test	Short and long term memory deficits	[37]
3-Quinuclidinyl benzilate	Competitive antagonist of cholinergic receptors	Step-through passive avoidance task, water maze test	Spatial memory deficits	[38]
Biperiden	Muscarinic antagonist	Verbal recognition task, Spatial memory task	Various memory deficits	[39]
Cisplatin	DNA damage, inflammation, mitochondrial dysfunction, apoptotic cell death, and oxidative damage	Water maze test	Spatial memory deficits	[40]
Phosphamidon	Inhibition of the activities of acetylcholinesterase	Passive avoidance and elevated plus maze	Short and long term memory	[41]
Tris-(2,3-ibromopropyl) Isocyanurate	Upregulation of inflammatory and oxidative stress markers, overexpression of pro-apoptotic proteins, down-expression of neurogenesis-related proteins in hippocampus, and hippocampal neurons damage	Forced swimming test, Morris water maze test	Spatial memory deficits	[42]
α-Synuclein	Oppose long-term potentiation and impair memory through a calcineurin-dependent mechanism	Fear conditioning	Long term memory deficit	[43]

## Abbreviations

6-OHDA	6-hydroxydopamine
Aβ	Amyloid beta
Ach	Acetylcholine
AChE	Acetylcholinesterase
AD	Alzheimer’s disease
Al	Aluminum
AMPA	α-amino-3-hydroxy-5-methyl-4-isoxazolepropionic acid
APP	Aβ precursor protein
As	Arsenic
ASK1	Apoptosis signal-regulating kinase 1
BBB	Blood-brain barrier
CaMK-II	Calcium/calmodulin-dependent protein kinase II
CBP	Cholinergic basal forebrain
Cdk5	Cyclin-dependent kinase 5
ChAT	Choline acetyltransferase
CNS	Central nervous system
cPLA2	Cytosolic phospholipase A2
CREB	cAMP response element-binding protein
CSF	Cerebrospinal fluid
Cu	Copper
DA	Dopamine
DAT	Dopamine transporter
DG	Dentate gyrus
GFAP	Glial fibrillary acidic protein
GSK-3	Glycogen synthase kinase-3
HAChT	Cholinergic nerve terminal
HAChT	High-affinity choline transport
HD	Huntington’s disease
Ibo	Ibotenic acid
iNOS	Inducible nitric oxide synthase
i.p	Intraperitoneal
JNK	c-jun N-terminal kinase
KA	Kainic acid
LTP	Long-term potentiation
MAO-B	Monoamine oxidase-B
MAPK	Mitogen-activated protein kinase
MFB	Medial forebrain bundle
MPTP	1-methyl-4-phenyl-1,2,3,6-tetrahydropyridine
MWM	Morris water maze
NBM	Nucleus basalis magnocellularis
NGF	Nerve growth factor
NMDA	*N*-methyl-d-aspartate
NOS	Nitric oxide synthase
OKA	Okadaic acid
Pb	Lead
PD	Parkinson’s disease
PFC	Prefrontal cortex
PI3K	Phosphoinositol 3-kinase
PP	Protein phosphatase
QA	Quinolinic acid
RACK1	Receptor for activated C-kinase 1
RIP	Ribosome-inactivating protein
ROS	Reactive oxygen species
SDAT	Sporadic dementia of Alzheimer’s type
SNpc	Substantia nigra pars compacta
STZ	Streptozotocin
TMT	Trimethyltin
VMAT2	Vesicular monoamine transporter
Zn	Zinc
